# Glycosaminoglycan Binding Facilitates Entry of a Bacterial Pathogen into Central Nervous Systems

**DOI:** 10.1371/journal.ppat.1002082

**Published:** 2011-06-23

**Authors:** Yung-Chi Chang, Zhipeng Wang, Lindsay A. Flax, Ding Xu, Jeffrey D. Esko, Victor Nizet, Miriam J. Baron

**Affiliations:** 1 Glycobiology Research and Training Center, University of California, San Diego, La Jolla, California, United States of America; 2 Department of Pediatrics, University of California, San Diego, La Jolla, California, United States of America; 3 Channing Laboratory, Brigham and Women's Hospital, Boston, Massachusetts, United States of America; 4 Children's Hospital, Boston, Massachusetts, United States of America; 5 Department of Cellular and Molecular Medicine, University of California, San Diego, La Jolla, California, United States of America; 6 Skaggs School of Pharmacy and Pharmaceutical Sciences, University of California, San Diego, La Jolla, California, United States of America; 7 Rady Children's Hospital, San Diego, California, United States of America; 8 Harvard Medical School, Boston, Massachusetts, United States of America; Harvard Medical School, United States of America

## Abstract

Certain microbes invade brain microvascular endothelial cells (BMECs) to breach the blood-brain barrier (BBB) and establish central nervous system (CNS) infection. Here we use the leading meningitis pathogen group B *Streptococcus* (GBS) together with insect and mammalian infection models to probe a potential role of glycosaminoglycan (GAG) interactions in the pathogenesis of CNS entry. Site-directed mutagenesis of a GAG-binding domain of the surface GBS alpha C protein impeded GBS penetration of the *Drosophila* BBB *in vivo* and diminished GBS adherence to and invasion of human BMECs *in vitro*. Conversely, genetic impairment of GAG expression in flies or mice reduced GBS dissemination into the brain. These complementary approaches identify a role for bacterial-GAG interactions in the pathogenesis of CNS infection. Our results also highlight how the simpler yet genetically conserved *Drosophila* GAG pathways can provide a model organism to screen candidate molecules that can interrupt pathogen-GAG interactions for future therapeutic applications.

## Author Summary

*Streptococcus agalactiae* (Group B *Streptococcus*, GBS) is a leading cause of meningitis in human newborn infants. The bacterial and host factors that allow this pathogen to cross the blood-brain barrier (BBB) and cause central nervous system (CNS) infection are not well understood. Here we demonstrate that GBS expresses a specific protein on its surface that can bind to sugar molecules known as glycosaminoglycans (GAGs) on the surface of brain capillary cells, initiating infection of the BBB. Fruit flies or mice genetically engineered to have reduced GAGs showed decreased dissemination of GBS into the brain tissues following experimental infection. Our results identify a role for bacterial-GAG interactions in the pathogenesis of newborn meningitis and highlight how the simpler yet genetically conserved fruit fly GAG biosynthetic pathways make the fruit fly a good model organism to screen candidate molecules that can interrupt pathogen-GAG interactions for future therapeutic applications.

## Introduction

Bacterial meningitis is one of the top ten causes of infection-related mortality worldwide [1]. Meningitis is particularly devastating in the newborn infant, and 20–50% of survivors can suffer permanent neurological sequelae including deafness, seizures, hydrocephalus, cerebral palsy and/or cognitive deficits [2]–[5]. The most common agent of neonatal bacterial meningitis in the United States, Europe and Asia is group B *Streptococcus* (GBS). In recent years, GBS has also emerged as a cause of serious infections including meningitis in nonpregnant adult populations, with an invasive disease incidence approaching that reported for the neonate [6]–[8]. In gaining access to the central nervous system (CNS), GBS reveals an ability to cross the blood-brain barrier (BBB), a specialized layer of brain microvascular endothelial cells (BMECs) that regulates macromolecular traffic to maintain biochemical homeostasis in brain tissues. BBB penetration by a bacterial pathogen reflects a complex interplay between host endothelium and microbial products [9]. The fundamental mechanisms by which GBS establishes CNS infection remain incompletely understood [10].

The plasma membrane of mammalian endothelial cells is prominently decorated with linear, negatively charged sugar chains known as glycosaminoglycans (GAGs). The sulfated GAGs, chondroitin/dermatan sulfate and heparan sulfate, occur as proteoglycans that consist of one or more GAG chains covalently linked to a core protein. Cells elaborate several membrane heparan sulfate proteoglycans (HSPGs) including syndecans and the glycosylphosphatidylinositol-linked glypicans. The heparan sulfate chains assemble by copolymerization of alternating residues of N-acetylglucosamine and glucuronic acid, which then can undergo variable sulfation and uronic acid epimerization. This process occurs in a non-template driven manner, resulting in sections of the chains with variably modified sugars of variable length interspersed with domains of unmodified residues. The modified regions make up binding sites for growth factors, membrane receptors, and extracellular matrix constituents, imparting to the chains important biological activities, such as roles in cell adhesion, receptor signaling, and leukocyte trafficking across endothelium [11]. The ability of an individual cell to interact with and respond to ligands appears to depend on the array of expressed proteoglycans and the pattern of modifications of the heparan sulfate chains [12]–[14]. Proteoglycan synthesis in mammals is a complex process involving a diverse array of gene products including core proteins, enzymes that initiate and elongate the polysaccharide chain, and enzymes that modify the polymer by sulfation and other processes [15], [16]. *Drosophila melanogaster* contains a repertoire of GAG structures similar to that of mammals [17] through a relatively small number of genes highly homologous to those found in mammals [18], [19].

A number of viral and bacterial pathogens functionally interact with cell surface GAGs [14]. Examples include the initial attachment phase of herpes simplex virus mediated through heparan sulfate [20], [21], *Mycobacterium tuberculosis* lung epithelial invasion through expression of a heparin-binding protein [22], and heparan sulfate-dependent attachment and entry of intestinal epithelial cells by *Listeria monocytogenes* [23]. We hypothesized that direct interactions with GAGs expressed on BMECs could influence the propensity of bloodborne bacteria to breach the BBB and produce CNS infection. In the specific case of GBS, a candidate ligand for this process exists in the surface-anchored Alpha C protein (ACP), which has the capacity to bind GAGs and promote bacterial entry into cervical epithelial cells *in vitro* [24]–[26].

*Drosophila* can serve as a useful model organism for analysis of mechanisms of bacterial pathogenesis and resistance [27]–[30]. We have recently shown that GBS infection can be established in *Drosophila*, and that overall mortality and bacterial loads are reduced in fly strains with diminished GAG expression [31]. In the present study, we use bacterial, *Drosophila*, and mouse mutants to achieve the aim of examining the role of bacterial-GAG interactions as mediators of BBB translocation and CNS infection. The study highlights an emerging recognition that specialized surface glial cells regulate the flow of substances into and out of the fly brain and present a functional equivalent of the BBB in *Drosophila* [32]–[35]. Our findings indicate that the specific heparan sulfate-binding properties of ACP promote BBB interactions and contribute to the establishment of GBS meningitis.

## Results

### GAG binding by ACP facilitates GBS dissemination into fly heads

**Figure 1:**
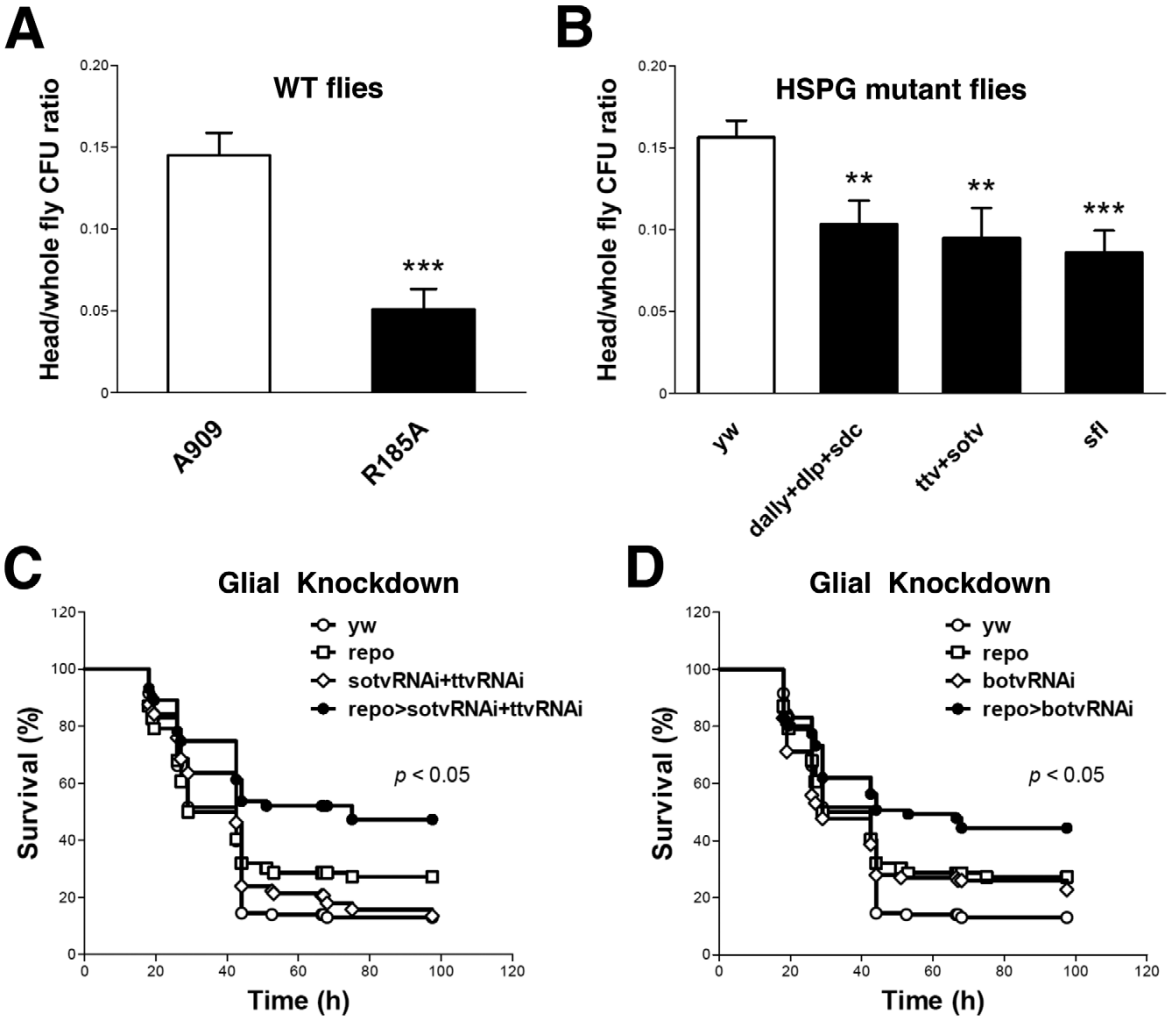
ACP-GAG binding promotes GBS invasion into fly heads; glial GAG polymerase knock-down decreases lethal infection. (A) The ratio of head/whole fly cfu in WT *yw* flies 24 h after infection with GBS mutant R185A (filled bar, *n* = 13 groups of 10 flies) was significantly lower than after infection with WT GBS strain A909 (open bar, *n* = 14 groups of 10 flies). (B) The ratios of head to whole fly cfu in HSPG mutant flies (filled bars) were lower than those in the WT control flies (open bar, *n* = 22 groups of 10 flies). The HSPG mutant flies studied here carry mutations in the genes encoding core proteins (*dally+dlp+sdc*, *n* = 15 groups of 10 flies), GAG polymerases (*ttv+sotv*, *n* = 12 groups of 10 flies) or the GAG NDST (*sfl*, *n* = 10 groups of 10 flies. Infected flies were heterozygotes from crosses of these mutants with *yw* flies, as homozygous mutants in these genes are nonviable. Each bar represents the mean and SEM. The student's *t*-test was used for statistical analysis in each comparison. ** *p*<0.01; *** *p*<0.001. *Repo*-Gal4 is a pan-glial Gal4 driver [66]. Flies expressing shRNA targeting both *ttv* and *sotv* (C) or *botv* alone (D) driven by *repo*-Gal4 show resistance to lethal A909 infection. Plots show a pool of data from at least 60 flies per sample. The genotypes of the flies used here are *repo*-Gal4/+ (repo), UAS-*sotv*RNAi/+;UAS-*ttv*RNAi/+ (sotvRNAi+ttvRNAi), UAS-*sotv*RNAi/+;UAS-*ttv*RNAi/*repo*-Gal4 (repo>sotvRNAi+ttvRNAi), UAS-*botv*RNAi/+ (botvRNAi) and UAS-*botv*RNAi/*repo*-Gal4 (repo>botvRNAi). The Gal4 and UAS lines were crossed with *yw* flies as controls. The survival curves of *yw*, Gal4 controls, UAS controls, and shRNA expressing groups are indicated as open circles, open squares, open diamonds, and filled circles respectively. A log rank test was used for statistical analysis in comparing shRNA-expressing groups and control groups.

We previously demonstrated that pricking the *Drosophila* thorax with a needle that had been dipped into a concentrated slurry of GBS leads to fly death [31]. To assess the nature of bacterial dissemination from the prick site, we performed histologic examination of GBS-infected *Drosophila*. As shown (Figure S1 *A* and *B*), wild-type (WT) GBS A909 spreads systemically after localized injection in the thorax. H&E stain reveals bacterial cocci in multiple sites, including muscle, fat, and the lining of the brain. These data indicate that tissue architecture is largely preserved despite widespread GBS dissemination during infection. The mutant GBS strain A909/R185A harbors a single amino acid change in ACP that significantly reduces GAG binding without affecting overall ACP structure, bacterial growth rate, or surface polysaccharide capsule expression [26], [31]. Following pinprick inoculation of an identical dose of the A909/R185A mutant into the fly thorax, bacteria disseminate less broadly from the local site of infection, and fewer cocci are visualized at the brain lining (Figure S1 *C* and *D*) To specifically address whether ACP-GAG binding promotes GBS dissemination into fly heads, we compared the bacterial burden (colony forming units, cfu) in the heads and bodies of WT flies after infection with WT GBS A909 and A909/R185A mutant. Flies infected with mutant bacteria had a significantly lower ratio of head cfu to total (head+body) cfu than those infected with the WT strain (Figure 1*A*; Supplemental Table S1), indicating that disruption of ACP-GAG binding decreases GBS penetration into the fly head.

### Impaired *Drosophila* GAG expression reduces GBS dissemination into fly heads

The *Drosophila* genome contains genes encoding the HSPG core proteins *dally*, *dally-like protein* [*dlp*] and *syndecan* [*sdc*]; three genes encoding heparan sulfate polymerases, *tout-velu* [*ttv*] (homolog of mammalian *Ext1*), *sister of ttv* [*sotv*] (homolog of *Ext2*) and *brother of ttv* [*botv*] (homolog of *Extl3*); and one gene encoding an N-deacetylase-N-sulfotransferase (*sulfateless* [*sfl*], a homolog of *Ndst1*). After chain initiation by Botv, polymerization occurs through a copolymerase complex consisting of Ttv and Sotv [36]. The GAG chains then undergo a series of modifications such as N-deacetylation and N-sulfation of N-acetylglucosamine residues by Sfl. Mutations in these genes lead to altered content or sulfation of heparan sulfate on all *Drosophila* heparan sulfate proteoglycans [37], [38].

To corroborate the role of GAG binding in GBS dissemination into the fly head, we used the pinprick method to establish infection in three different HSPG mutant fly strains deficient in membrane HSPG core proteins (*dally+dlp+sdc*), heparan sulfate polymerases (*ttv+sotv*), or NDST (*sfl*), respectively. After infection with WT GBS A909, all three HSPG mutant fly strains exhibited lower head/total body cfu ratios than did the WT control flies (Figure 1*B*; Supplemental Table S1). The extent of reduction in head/total body cfu probably underestimates the importance of HSPGs in this model since each of the host mutations had to be tested as heterozygotes due to the requirement for HSPGs in fly embryogenesis.

We next sought to specifically link the observed GAG-dependent phenotype to glial cells that form tight junctions and restrict paracellular diffusion between the fly circulatory system and brain, thereby representing the *Drosphila* BBB equivalent [33]. The glial cell-specific driver *repo*-Gal4 was used to suppress polymerase expression in fly BBB cells through *in vivo* shRNA knockdown of *ttv*, *sotv*, and *botv*. After infection with WT GBS A909, flies with glial cell-specific knockdown of either *botv* alone, or both *ttv* and *sotv* displayed higher survival rates than the corresponding repo and Gal4-UAS controls (Figure 1 *C* and *D*).

#### GAG binding by GBS ACP promotes human BMECs adherence and invasion

**Figure 2:**
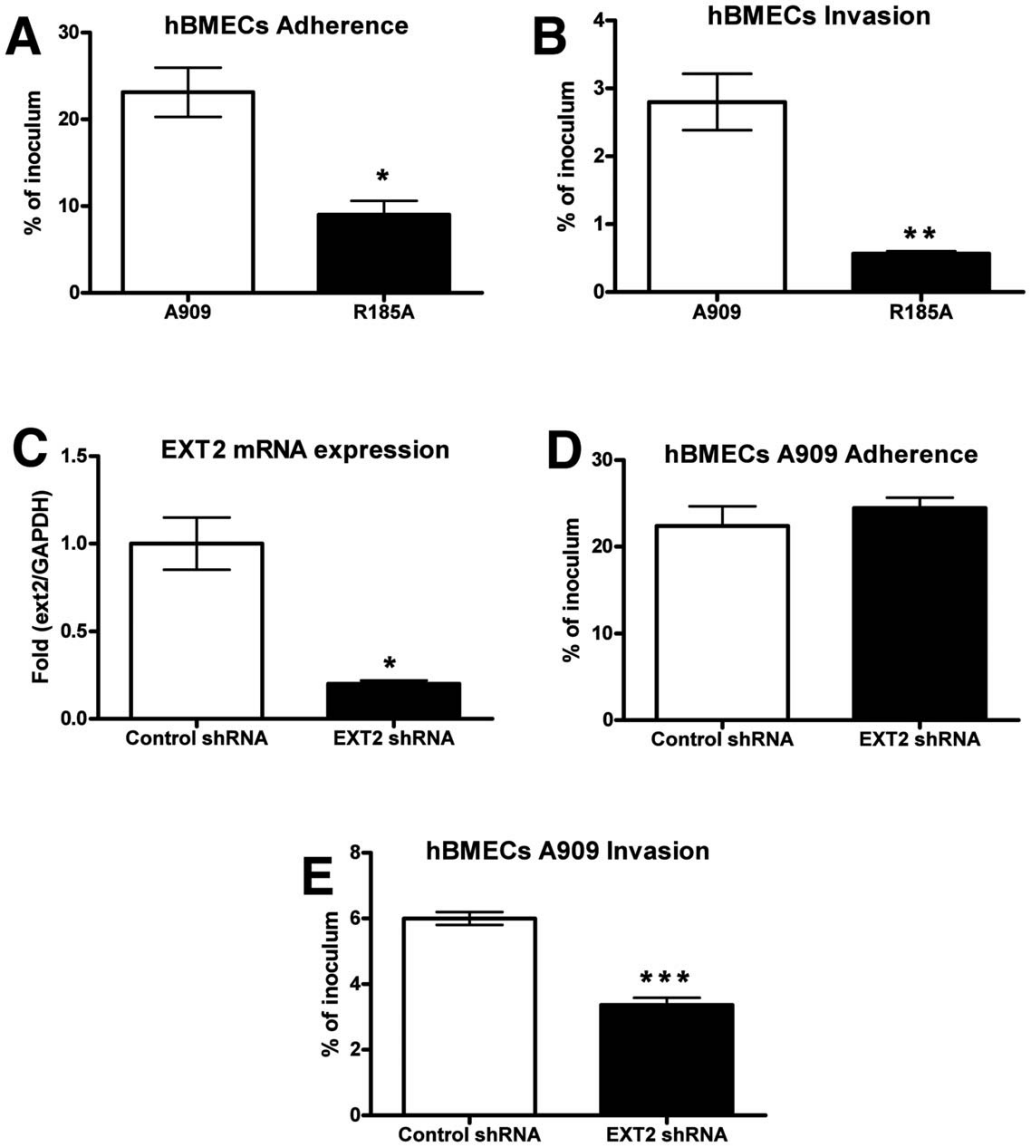
Contribution of GAG-binding activity of GBS to adherence and invasion of hBMECs. For adherence (A), bacteria were enumerated after 30 min of incubation, whereas invasion (B) was quantified after 2 h of incubation with human brain microvascular endothelial cells (hBMECs) and 2 h of incubation with antibiotics to kill extracellular bacteria. (C) Knockdown of EXT2 expression by RNA interference. hBMECs were infected with lentiviruses carrying control shRNA or EXT2 targeting shRNA and the knockdown efficiency was determined by quantitative RT-PCR. GAPDH was used as an internal control. Attenuation of A909 invasion (E) but not adherence (D) after knocking down EXT2 expression in hBMECs. Adherence and invasion assays were performed in triplicate and repeated three times with similar results, and representative experiments are shown. Statistical analysis was performed by Student's *t* test and error bars SEM. * *p*<0.05; ** *p*<0.01; *** *p*<0.001.

Immortalized human BMECs have provided a widely used and well-defined *in vitro* model for understanding microbial interactions with the human BBB [9], [39]. Previous studies have shown that fetal or adult primary BMECs expressed high levels of heparan and chondroitin sulfates [40], supporting earlier conclusions that proteoglycans are richly expressed on the surface of primary human endothelial cells derived from all age groups, including the fetus [41]–[43]. We found that WT GBS A909 efficiently adhered to and invaded hBMECs; however the A909/R185A mutant with decreased GAG-binding ability showed significantly reduced adhesion and invasion of these cells (Figure 2 *A* and *B*). Similar results were obtained when infection and invasion were measured in A549 lung adenocarcinoma cells (Figure S2 *A* and *B*). To further establish a role for GAG binding in GBS interaction with hBMECs, we used RNA interference to silence the expression of EXT2. The efficiency of silencing was over 80% by qRT-PCR analysis of mRNA from hBMECs infected with EXT2 shRNA lentiviruses compared to hBMECs infected with a control lentivirus (Figure 2*C*). Although no apparent difference in WT GBS A909 adherence to control vs. EXT2 knockdown hBMECs was discerned (Figure 2*D*), GBS invasion of hBMECs following EXT2 knockdown was significantly decreased (Figure 2*E*).

### Reduced GAG expression diminishes GBS BBB penetration in a murine meningitis model

**Figure 3:**
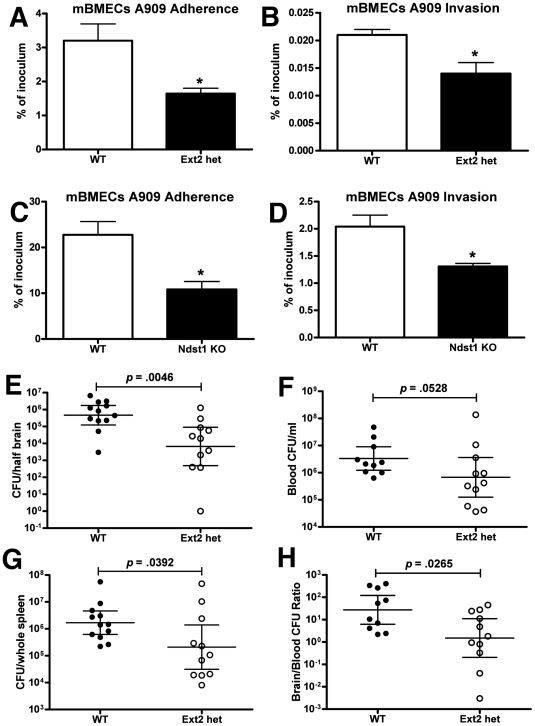
GAG contributes to blood-brain barrier penetration in mice *in vivo*. Attenuated GBS adhesion (A, C) and invasion (B, D) to primary mBMECs from Ext2 heterozygous mice (ext2 hets) and Ndst1-deficient mice. For adherence, bacteria were enumerated after 30 min of incubation, whereas invasion was quantified after 2 h of incubation with mBMECs and 2 h of incubation with antibiotics to kill extracellular bacteria. Comparison of bacterial counts (expressed in cfu) recovered from the brain (E), blood (F), and spleen (G) of WT mice and Ext2 hets 24 h after intravenous challenge with 10^8^ cfu of A909. (H) Brain bacterial counts were corrected for blood contamination (brain/blood ratio) using the blood concentration and a conservative estimate of the mouse cerebral blood volume (2.5 ml per 100 g tissue). Results pool the data from two independent experiment with final numbers of *n* = 12 for WT and *n* = 11 for Ext2 het. Each circle denotes 1 mouse. Statistics analysis was performed by Student's *t* test (A–D) or Mann-Whitney test (E–H). *p<0.05.

Mice heterozygous for *Ext2* provide a functional *in vivo* model in which the chain length of GAGs expressed by the host is significantly reduced [44]. To confirm the utility of this model for analysis of GBS-BBB interactions, we isolated and cultured primary murine BMECs (mBMECs) from *Ext2* ^+/−^ mice (Ext2 hets) and WT littermate controls and infected them *ex vivo* with WT GBS A909. We documented an approximate 40% reduction in heparan sulfate content of endothelium isolated from these Ext2 het mice vs. WT controls (Figure S3). GBS adherence and invasion were significantly reduced in mBMECs from Ext2 hets compared to those isolated from WT mice (Figure 3 *A* and *B*). Several factors may explain why adherence was altered in Ext2 het mBMECs but not in Ext2-silenced hBMECs, including differences in residual GAG quantity or structure or overall surface charge, other effects of using transformed vs. primary cells, or species-specific effects. Of note, Ext2-silencing was associated with reduced hBMEC growth rate, while Ext2 het mBMECs grew similarly to WT mBMECs.

Moreover, impaired GBS adherence and invasion was also observed in Ndst1-deficient mBMECs that exhibit overall reduction in sulfation of the chains (Figure 3 *C* and *D*) [36], [37]. Comparable results were obtained in Ndst1-deficient mouse lung endothelial cells (Figure S2 *C* and *D*). To explore a potential contribution of mammalian GAG expression to GBS BBB penetration *in vivo*, Ext2 hets and WT littermate controls were infected intravenously with WT GBS A909 and sacrificed 24 h later. Significantly fewer bacterial cfu (9-fold decrease) were recovered from the brains of GBS-infected Ext2 hets compared to brains of WT controls (Figure 3*E*) and some animals showed dramatic 10^3^–10^4^ fold reduction in brain cfu. In contrast, lesser decreases in bacterial cfu were observed in the blood (Figure 3*F*) and spleen (Figure 3*G*) of Ext2 hets. Consequently, the mean ratio of brain∶blood cfu in Ext2 hets (10.6) was significantly lower than that observed in WT controls (116.0) (Figure 3*H*). In a small pilot experiment, we noted a trend of reduced CFU in the lungs of Ext2 hets compared to WT mice (Figure S4), suggested that the finding may extend to other endothelium, but such conclusions require future corroboration. Bone-marrow derived macrophages from Ext2 hets and WT controls did not differ in assays of phagocytosis, total bacterial killing, intracellular bacterial killing, or release of tumor necrosis factor-alpha when challenged with GBS *ex vivo* (Figure S5), indicating that reduced GAG expression does not globally compromise phagocyte innate immune function. In sum, the reduction of GAG expression resulting from EXT2 heterozygosity diminishes GBS interactions with BMECs *in vitro* and decreases GBS CNS entry *in vivo*.

## Discussion

Our combined analyses in the *Drosophila* and mammalian systems indicate that GBS interaction with GAGs promotes BBB attachment and bacterial entry into the CNS. The GAG binding property of the surface-anchored ACP is one contributor to this invasive phenotype. Meningitis is a dangerous complication of neonatal GBS infection and understanding the predilection of the pathogen to breach the BBB is a critical goal of molecular pathogenesis investigations. To date, a number GBS factors have been shown to promote its interaction with human BMECs *in vitro*, including fibrinogen adhesin FbsA [38], laminin-binding protein Lmb [45], major pilus backbone subunit PilB [46], lipoteichoic acid anchoring enzyme IagA [47], and the serine-rich repeat 1 glycoprotein Srr1 [48]. In the case of IagA, Srr1, and the hBMEC-disrupting GBS ß-hemolysin/cytolysin, a contribution to meningitis in the murine model has further been demonstrated [47]–[49]. However, the present study is the first to genetically manipulate the host to identify the corresponding host receptor molecules (GAGs) that the GBS virulence factor (ACP) exploits to adhere to and invade the BBB endothelium.

Mammalian heparin sulfate chain polymerization reactions are carried out by the exostosin proteins EXT1 and EXT2 [50], [51] and subsequent modification of the chains by the bifunctional NDSTs. We found decreased GBS invasion in both hBMECs with shRNA-reduced EXT2 levels (Figure 2*E*) and primary Ext2 heterozygous mBMECs (Figure 3*B*). In additional studies, we found reduced GBS adherence to and invasion of Ndst1-deficient mBMECs (Figure 3 *C* and *D*) and lung endothelial cells (Figure S2 *C* and *D*). Adherence and invasion of the A909/R185A mutant to A549 human lung epithelial cells were also markedly attenuated compared to the WT GBS A909 (Figure S2 *A* and *B*). Thus the GAG-ACP interaction may contribute to GBS penetration of a broader spectrum of host cell barriers, and GAG expression patterns may determine the nature and efficiency of bacterial dissemination during infection. Moreover, our findings suggest that the chain lengths and negative charges on the GAG chains are both important to provide the binding forces between ACP and GAGs. These findings are consistent with prior data demonstrating that specific positively charged residues of ACP are required for full ACP-GAG binding affinity [26]. Whether ACP binds to a specific sequence of sulfated sugars within heparan sulfate remains to be determined. The availability of mutants altered in 2-O-sulfation of uronic acids, and 3-O- and 6-O-sulfation of glucosamine residues will allow further studies on the structural specificity of the interaction in flies [52], [53] and in mice [54], [55].

Our parallel findings in flies and in mammals both *in vitro* and *in vivo* support the relevance of a *Drosophila* infection model for the study of human CNS infections. Others have reported that the *Drosophila* humoral/CNS barrier conserves the xenobiotic exclusion properties of vertebrate vascular endothelium. Specifically, the exclusion process is mediated in part by a fly ATP binding cassette (ABC) transporter, Mdr65, that functions similarly to mammalian xenobiotic blood-brain barrier transporters. Thus, CNS chemoprotection involves both conserved molecular structures and functionally analogous anatomic spaces that together promote CNS selective drug partition [34]. Our data extend these findings by demonstrating that CNS penetration of microorganisms may also occur via conserved molecular structures (GAGs), and that these structures can be studied effectively in a *Drosophila* infection model.

Many microorganisms display GAG-binding ability, including the two leading bacterial pathogens associated with meningitis in older children and adults: *Streptococcus pneumoniae* utilizes heparin, heparan sulfate and chondroitin/dermatan sulfate in colonization of respiratory mucosal epithelial cells [56], and *Neisseria meningitidis* surface protein OpC binds HS proteoglycans to initiate epithelial cell invasion [57]. Sulfated GAGs also promote adherence of the veterinary meningeal pathogen *Haemophilus somnus* to bovine BMEC *in vitro* [58]. However, each of these GAG-binding interactions has uncertain functions in the pathogenesis of CNS infection that could be difficult to pinpoint because of functional redundancy of bacterial adhesins/invasins and the challenges of manipulating GAG structure/expression in complex mammalian host systems. The importance of positively charged residues is a common theme among GAG-binding proteins. For example, the C-terminal regions of mycobacterial heparin-binding hemagglutinin [59] and histone-like Hlp protein [60] contain Arg/Lys-rich repeats important for heparin binding. In some instances, common heparin-binding motifs, such as BBXB, BBBXXB (B representing a basic amino acid residue), and a 20 Å spacing of basic residues have been reported [61], [62]. Positively charged residues of ACP that were confirmed to contribute to GAG binding by site-directed mutagenesis were R172, R185 and K196; two of these (R185 and K196) are completely conserved in other members of the alpha-like protein (Alp) including Rib, Alp1, Alp2, Alp3 and Alp4 of GBS and R28 of group A *Streptococcus* [26].

The *Drosophila* infection model offers the advantages of a simpler genome and well-developed molecular approaches that will facilitate interrogation of host-pathogen GAG-binding interactions, and allow testing of candidate inhibitors of these interactions with an eye toward future therapeutic applications. The design of inhibitors targeting particular pathogen virulence mechanisms represents an attractive strategy in the era of increasing resistance to conventional antibiotics. In attempting to block bacterial-GAG interactions, a major limitation to competitive inhibition by heparin itself is its potency as an anticoagulant and the risk of hemorrhagic complications. However, synthetic low-molecular weight heparins or analogs devoid of anticoagulant activity could be contemplated in this context as potential adjunctive agents for infectious disease therapeutics.

## Materials and Methods

### Ethics statement

This study was carried out in strict accordance with the recommendations in the Guide for the Care and Use of Laboratory Animals of the National Institutes of Health. The protocol was approved by the Institutional Animal Care and Use Committee of the University of California, San Diego (Animal Welfare Assurance Number: A3033-01). All efforts were made to minimize suffering of animals employed in this study.

### Bacteria and cell lines

The ACP-expressing human serotype Ia GBS neonatal isolate, A909, was used in this study. A909/R185A is an A909 point mutant strain that has an ACP variant with diminished GAG binding affinity [26]. GBS were grown in Todd-Hewitt broth (THB, Difco) at 37°C. SV40 large T antigen immortalized human brain endothelial cell line (hBMEC) was obtained from Kwang Sik Kim (Johns Hopkins University, Baltimore, MD). hBMECs were maintained in RPMI 1640 medium (Invitrogen) supplemented with 10% FBS, 10% NuSerum (BD), and 1% MEM nonessential amino acids, and were incubated at 37°C in 5% CO_2_.

### Mice and murine primary brain microvascular endothelial cell isolation

Murine brain microvascular endothelial cells (mBMEC) were isolated from cerebral cortex as described [63], except that cells were selected with 5 µg/ml puromycin for 4 days in low-glucose DMEM medium supplemented with 20% FBS (Atlanta Biologicals), 50 µg/ml endothelial growth supplement (BTI), 50 µg/ml heparin, nonessential amino acids, penicillin and streptomycin. Cell purity was higher than 98% as assessed by blood endothelial markers including CD31, CD34, CD105 and CD166. Primary cells were cultured for 5 days and passaged once for experiments. Lung microvascular endothelial cells were isolated as described previously [64]. *Ext2* ^+/−^ mice were described previously [44]. Endothelial cells lacking Ndst1 were derived from *Ndst1* ^f/f^ *Tie2Cre* ^+^ mice [64], [65].

### Fly infections

In infection experiments, bacteria were grown to an optical density at 650 nm of 0.3, and then concentrated 10-fold to approximately 2×10^9^ cfu/ml; the exact bacterial concentration was confirmed in each experiment. Adult male flies (2 to 5 days old) were anesthetized with CO_2_ and then pricked in the dorsal thorax underneath the wing with a fine needle previously dipped in THB broth or a concentrated solution of GBS in THB. After infection, flies were incubated at 29°C in vials with food, and fly survival was monitored over the following 4 days. Cumulative survival curves were derived, and the median survival time for each group was determined using Kaplan-Meier survival analysis. A log rank test was performed to compare survival curves.

To determine the bacterial load in fly heads and bodies at 24 h after infection, flies were placed on ice, and fly heads were separated from fly bodies by a sterile surgical blade. The heads and bodies of 10 flies per group were homogenized in 500 µl and 1000 µl of phosphate-buffered saline (PBS) with 0.025% Triton X-100 respectively. The homogenates were diluted in series (usually 10^−1^ to 10^−3^), and the dilutions were plated on THB plates and incubated overnight at 37°C for cfu counting.

### Adherence and invasion assay

BMECs were split into 24-well plates and allowed to grow to confluence for 48 h prior to assays, to ensure similar cell numbers for each experiment. Confluent monolayers were incubated with log-phase grown bacteria at an MOI of 1 or 10, and centrifuged at 1600 rpm for 5 min to initiate contact. After 2 h incubation, the monolayers were washed, and 1 mL of media containing 100 µg of gentamicin and 5 µg of penicillin G was added for an additional 2 h. After washing, monolayers were disrupted by 0.025% Triton X-100, and the number of invasive bacteria was quantified by serial dilution plating. To assess the level of surface-adherent (total cell-associated) bacteria, bacteria were quantified after 30 min of incubation without addition of antibiotics. All cellular adherence and invasion assays were performed in triplicate and repeated at least 2–3 times.

### RNA interference

Human ext2 lentiviral shRNA construct (TRCN0000039849) was purchased from Open Biosystems. The resulting viruses were produced by cotransfection of 293T cells with the shRNA plasmid and packaging vectors (Open Biosystems) according to the vendor's instruction. The knockdown efficiency was determined by qRT-PCR analysis of ext2 expression. Primers used for qRT-PCR were EXT2 forward, 5′-AAGCACCAGGTCTTCGATTACC-3′ and reverse, 5′-GAAGTACGCTTCCCAGAACCA-3′. and GAPDH forward 5′-GAAGGTGAAGGTCGGAGTCAACG-3′ and reverse 5′-TCCTGGAGGATGGTGATGGAAT-3′.

### Mouse infection model

Ext2 heterozygous and littermate controls (10–12 weeks) were injected via the tail vein with 10^8^ cfu A909. Twenty-four h after injection, samples of blood, brain/meninges, and spleen were collected aseptically from mice after euthanasia. Bacterial counts in blood and tissue homogenates were determined by plating serial dilutions. Bacterial counts in brain and spleen samples were corrected for differences in organ weight. Brain bacterial counts were corrected for blood contamination using the blood concentration and a conservative estimate of the mouse cerebral blood volume [47]. In a pilot experiment, additional Ext2 heterozygous and littermate controls were injected intravenously with 1×10^8^ cfu of WT GBS and lungs harvested at 24 h for cfu determination. The significance of differences between treatment groups was determined using the unpaired Student *t* test.

## Supporting Information

Figure S1 Wild-type (OreR) *Drosophila* were pricked in the thorax with a needle dipped in a concentrated slurry (approx 2×10^9^ cfu/ml) of GBS wild-type strain A909 or A909/R185A, then incubated at 29°C in vials with food for 24 h, fixed in formalin, sectioned, and stained with H&E. No organisms were seen in control samples that were pricked with a needle dipped in sterile THB alone. (A) A909 infiltrating fat and muscle tissue, (B) A909 in clumps lining the brain (arrow). Tissue architecture is largely preserved despite widespread GBS dissemination. R185A spreads minimally if at all to (C) muscle and (D) CNS.
(TIF)

Figure S2 ACP-sulfated GAG interaction promotes GBS adherence and invasion of lung epithelial cells. GAG-binding diminished GBS mutant (R185A, filled bars) showed reduced adherence (A) and invasion (B) of A549 WT human lung epithelial cells compared to WT GBS strain A909 (open bars). Compared to wild-type cells, adherence (C) and invasion (D) of murine Ndst1-deficient lung endothelial cells (mLECs) by A909 (filled bars) were reduced. R185A results (open bars) are also shown. For adherence, bacteria were enumerated after 30 min of incubation, whereas invasion was quantified after 2 h of incubation with cells and 2 h of incubation with antibiotics to kill extracellular bacteria. Adherence and invasion assays were performed in triplicate and repeated three times with similar results, and representative experiments are shown. Statistical analysis was performed by Student's *t* test and error bars represent SEM. * *p*<0.05; ** *p*<0.01; *** *p*<0.001.
(TIF)

Figure S3 Reduction of heparan sulfate in lung endothelial cells isolated from Ext2+/− mice. Radiolabeled (^35^S) glycosaminoglycans were purified by column chromatography, proteinase- and chondroitinase-treated and heparan sulfate content of eluted material calculated by ^35^S content.
(TIF)

Figure S4 Pilot experiment comparing lung cfu from WT vs. Ext2 het mice 24 h following intravenous infection. Statistics analysis was performed by Mann-Whitney test.
(TIF)

Figure S5 WT and Ext2 het macrophages exhibited similar responses to GBS stimulation. Murine bone marrow-derived macrophages (MBDMs) were used for *in vitro* assays to examine phagocytosis (A), bacterial killing (B and C; WT, filled bars; Ext2 het, open bars) and TNF-α secretion (D; A909, filled bars; R185A, open bars) after GBS infection. For phagocytosis, A909 was added to MBDMs at MOI = 5 for 30 min, followed by 2 h of incubation with antibiotics to kill extracellular bacteria. Intracellular survival of A909 was performed at multiplicity of infection = 5 bacteria/cell. MBDMs were incubated with A909 for 60 min, followed by 2 h of incubation with antibiotics to kill extracellular bacteria. Intracellular colony forming units (CFU) were enumerated by the lysis of cells at the indicated time points. Total bacterial killing was performed at MOI = 0.1. For TNF-α assay, MBDMs were stimulated with A909 at MOI = 5 for 30 min, followed by 24 h of incubation with antibiotics. Supernatant was collected for ELISA assay to determine the TNF-α concentration.
(TIF)

Table S1 Head and body CFU counts in *Drosophila melanogater* infected with group B *Streptococcus*. This table provides all the primary data used in the calculations of Figure 1 *A* of the main text.
(DOC)

Text S1 Supplemental methods describing the measurement of heparan sulfate in primary mouse lung microvascular endothelial cells.
(DOC)
