# Supplementary figures and images for: Glycosaminoglycan Binding Facilitates Entry of a Bacterial Pathogen into Central Nervous Systems
Source: PLoS Pathog. 2011 Jun 23;7(6):e1002082. doi: 10.1371/journal.ppat.1002082 (PMC3121876; doi:10.1371/journal.ppat.1002082)

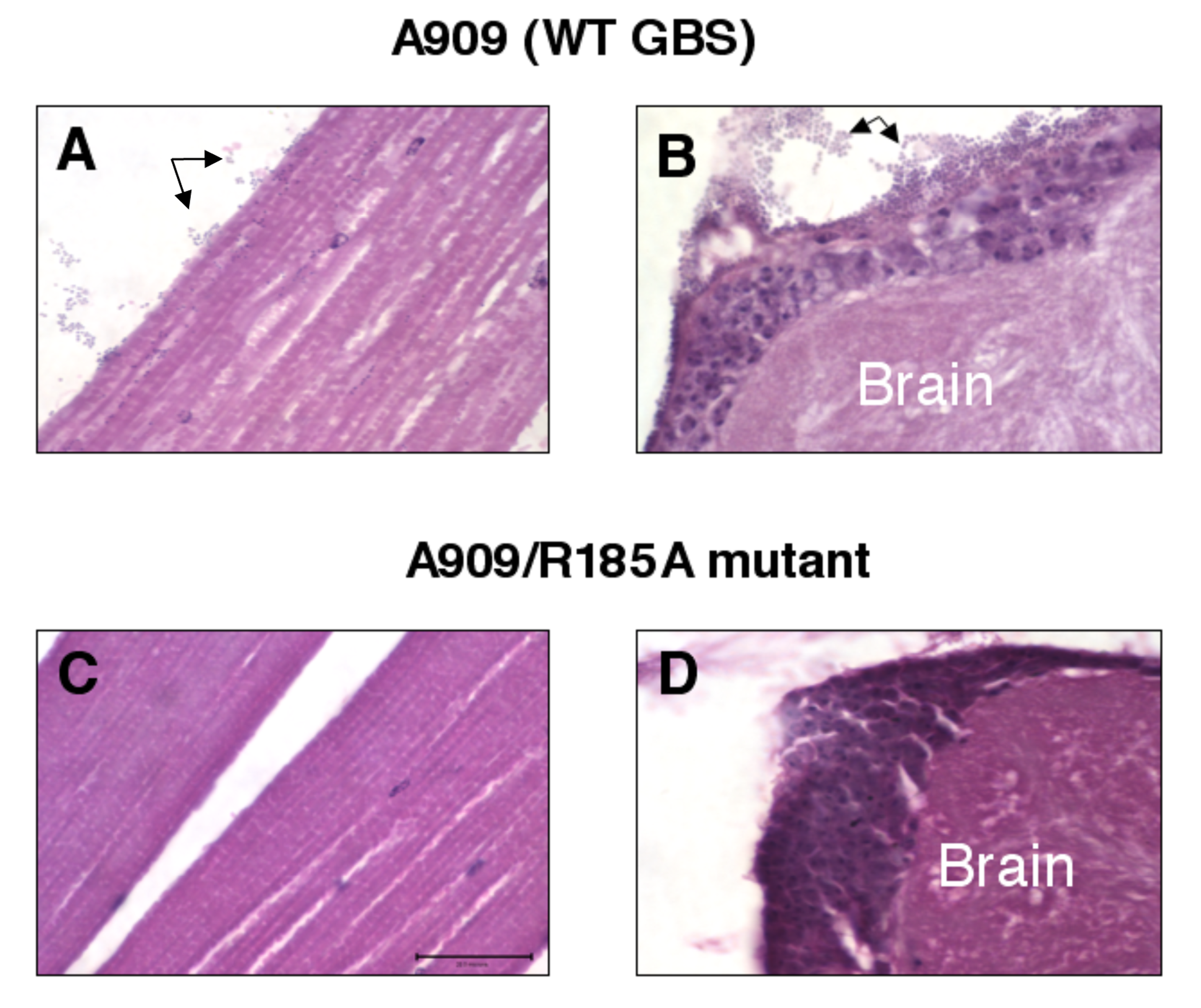

Supplement: Figure S1 — Wild-type (OreR) Drosophila were pricked in the thorax with a needle dipped in a concentrated slurry (approx 2×109 cfu/ml) of GBS wild-type strain A909 or A909/R185A, then incubated at 29°C in vials with food for 24 h, fixed in formalin, sectioned, and stained with H&E. No organisms were seen in control samples that were pricked with a needle dipped in sterile THB alone. (A) A909 infiltrating fat and muscle tissue, (B) A909 in clumps lining the brain (arrow). Tissue architecture is largely preserved despite widespread GBS dissemination. R185A spreads minimally if at all to (C) muscle and (D) CNS. (TIF) [file ppat.1002082.s001.tif]

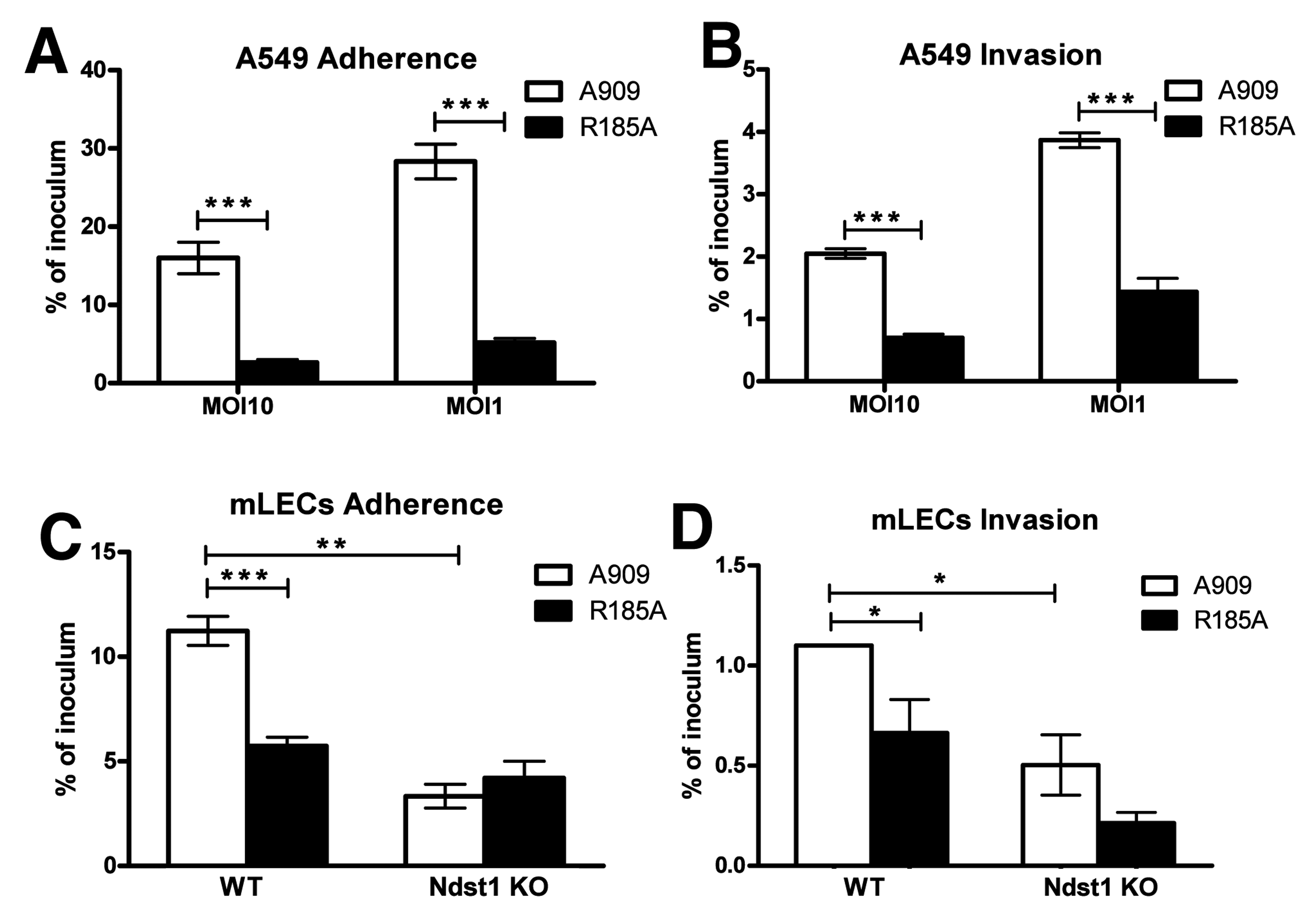

Supplement: Figure S2 — ACP-sulfated GAG interaction promotes GBS adherence and invasion of lung epithelial cells. GAG-binding diminished GBS mutant (R185A, filled bars) showed reduced adherence (A) and invasion (B) of A549 WT human lung epithelial cells compared to WT GBS strain A909 (open bars). Compared to wild-type cells, adherence (C) and invasion (D) of murine Ndst1-deficient lung endothelial cells (mLECs) by A909 (filled bars) were reduced. R185A results (open bars) are also shown. For adherence, bacteria were enumerated after 30 min of incubation, whereas invasion was quantified after 2 h of incubation with cells and 2 h of incubation with antibiotics to kill extracellular bacteria. Adherence and invasion assays were performed in triplicate and repeated three times with similar results, and representative experiments are shown. Statistical analysis was performed by Student's t test and error bars represent SEM. * p<0.05; ** p<0.01; *** p<0.001. (TIF) [file ppat.1002082.s002.tif]

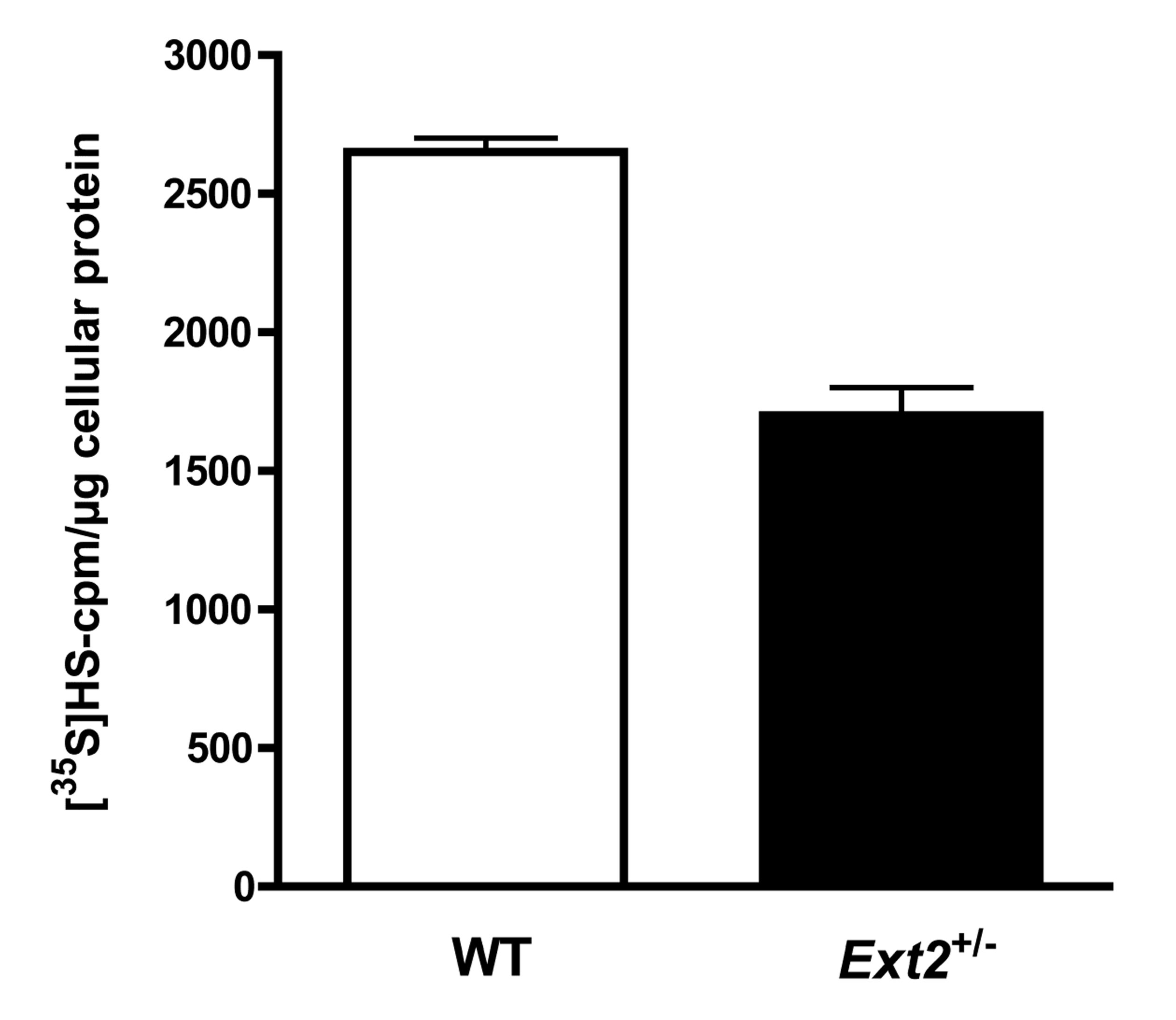

Supplement: Figure S3 — Reduction of heparan sulfate in lung endothelial cells isolated from Ext2+/− mice. Radiolabeled (35S) glycosaminoglycans were purified by column chromatography, proteinase- and chondroitinase-treated and heparan sulfate content of eluted material calculated by 35S content. (TIF) [file ppat.1002082.s003.tif]

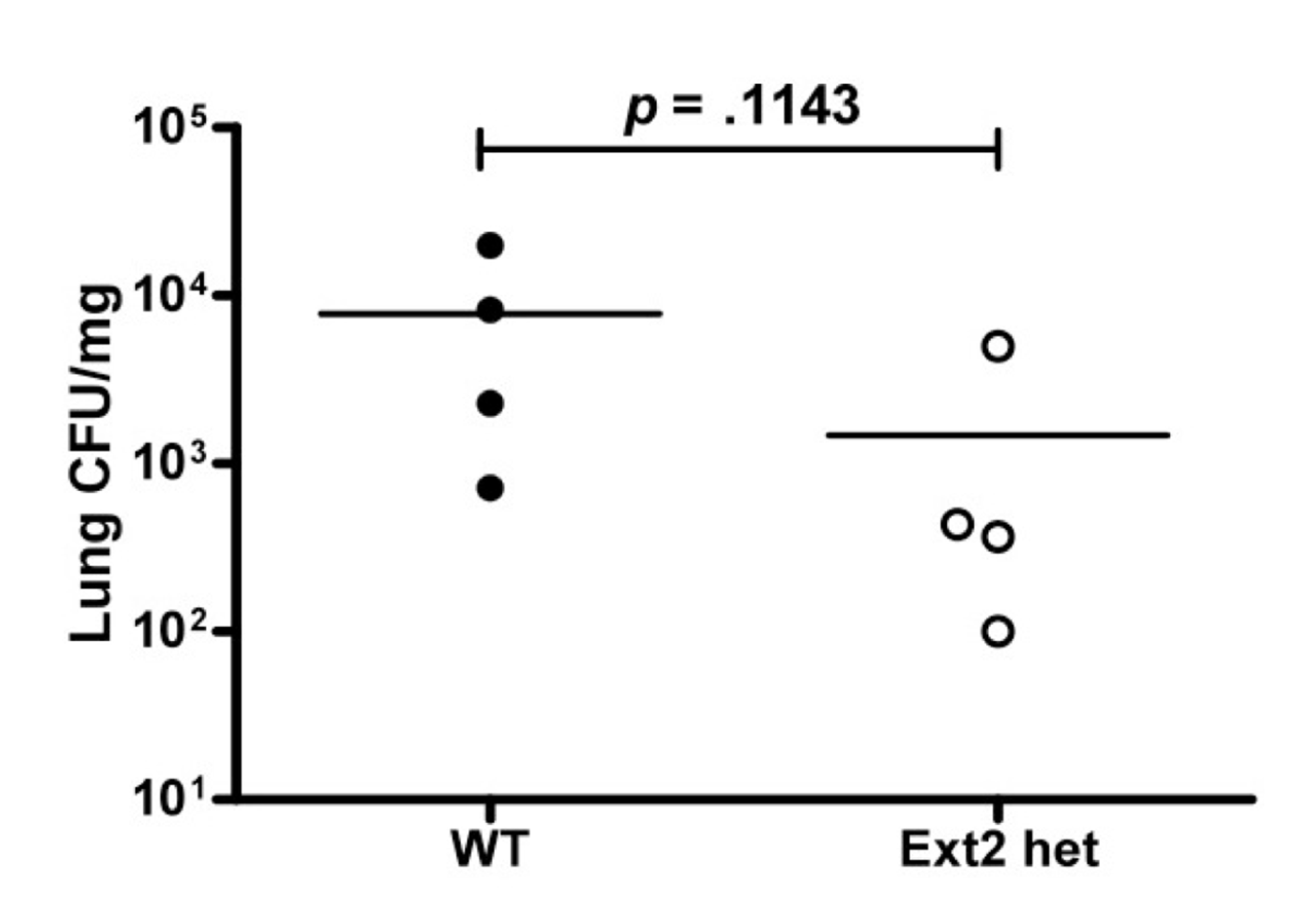

Supplement: Figure S4 — Pilot experiment comparing lung cfu from WT vs. Ext2 het mice 24 h following intravenous infection. Statistics analysis was performed by Mann-Whitney test. (TIF) [file ppat.1002082.s004.tif]

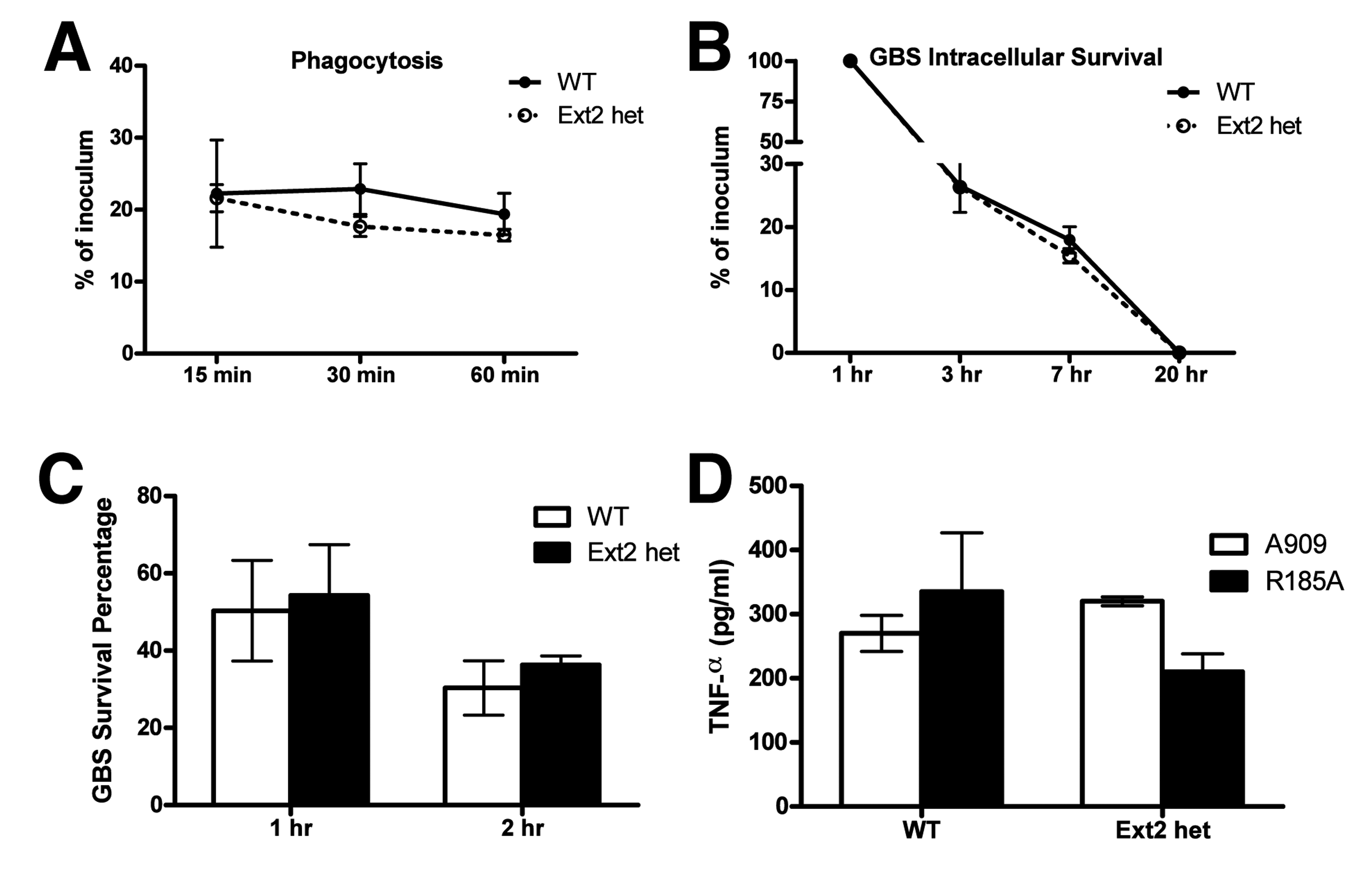

Supplement: Figure S5 — WT and Ext2 het macrophages exhibited similar responses to GBS stimulation. Murine bone marrow-derived macrophages (MBDMs) were used for in vitro assays to examine phagocytosis (A), bacterial killing (B and C; WT, filled bars; Ext2 het, open bars) and TNF-α secretion (D; A909, filled bars; R185A, open bars) after GBS infection. For phagocytosis, A909 was added to MBDMs at MOI = 5 for 30 min, followed by 2 h of incubation with antibiotics to kill extracellular bacteria. Intracellular survival of A909 was performed at multiplicity of infection = 5 bacteria/cell. MBDMs were incubated with A909 for 60 min, followed by 2 h of incubation with antibiotics to kill extracellular bacteria. Intracellular colony forming units (CFU) were enumerated by the lysis of cells at the indicated time points. Total bacterial killing was performed at MOI = 0.1. For TNF-α assay, MBDMs were stimulated with A909 at MOI = 5 for 30 min, followed by 24 h of incubation with antibiotics. Supernatant was collected for ELISA assay to determine the TNF-α concentration. (TIF) [file ppat.1002082.s005.tif]
