# Supplementary material for: Glycosaminoglycan Binding Facilitates Entry of a Bacterial Pathogen into Central Nervous Systems
Source: PLoS Pathog. 2011 Jun 23;7(6):e1002082. doi: 10.1371/journal.ppat.1002082 (PMC3121876; doi:10.1371/journal.ppat.1002082)
Supplement: Table S1 — Head and body CFU counts in Drosophila melanogater infected with group B Streptococcus. This table provides all the primary data used in the calculations of Figure 1 A of the main text. (DOC) [file ppat.1002082.s006.doc]

| GBS strain | A909 | A909 |  |  | R185A | R185A |  |  |
| --- | --- | --- | --- | --- | --- | --- | --- | --- |
| fly strain | yw | yw |  |  | yw | yw |  |  |
|  | head cfu/group of 10 flies | body cfu/group of 10 flies | head/body | head/whole fly | head cfu/group of 10 flies | body cfu/group of 10 flies | head/body | head/whole fly |
|  | 62000 | 499000 | 0.124248 | 0.110517 | 1000 | 17800 | 0.05618 | 0.053191 |
|  | 560000 | 3160000 | 0.177215 | 0.150538 | 250 | 8000 | 0.03125 | 0.030303 |
|  | 342500 | 3470000 | 0.098703 | 0.089836 | 2800 | 44300 | 0.063205 | 0.059448 |
|  | 1215000 | 6140000 | 0.197883 | 0.165194 | 200 | 18900 | 0.010582 | 0.010471 |
|  | 705000 | 4590000 | 0.153595 | 0.133144 | 425 | 11100 | 0.038288 | 0.036876 |
|  | 765000 | 5020000 | 0.15239 | 0.132239 | 50 | 8150 | 0.006135 | 0.006098 |
|  | 30250 | 341000 | 0.08871 | 0.081481 | 11000 | 164500 | 0.066869 | 0.062678 |
|  | 152750 | 940000 | 0.1625 | 0.139785 | 66250 | 325000 | 0.203846 | 0.169329 |
|  | 95500 | 1110000 | 0.086036 | 0.07922 | 19500 | 237500 | 0.082105 | 0.075875 |
|  | 615000 | 1580000 | 0.389241 | 0.280182 | 1000 | 45000 | 0.022222 | 0.021739 |
|  | 200000 | 890000 | 0.224719 | 0.183486 | 0 | 20000 | 0 | 0 |
|  | 575000 | 3100000 | 0.185484 | 0.156463 | 1500 | 38500 | 0.038961 | 0.0375 |
|  | 620000 | 3440000 | 0.180233 | 0.152709 | 1500 | 14000 | 0.107143 | 0.096774 |
|  | 1090000 | 5160000 | 0.21124 | 0.1744 |  |  |  |  |

Data for Figure 1 *B*

| GBS strain | A909 | A909 |  |  | A909 | A909 |  |  |
| --- | --- | --- | --- | --- | --- | --- | --- | --- |
| fly strain | yw | yw |  |  | dally+dlp+sdc | dally+dlp+sdc | |  |
|  | head cfu/group of 10 flies | body cfu/group of 10 flies | head/body | head/whole fly | head cfu/group of 10 flies | body cfu/group of 10 flies | head/body | head/whole fly |
|  | 560000 | 3160000 | 0.177215 | 0.150538 | 193000 | 1000000 | 0.193 | 0.161777 |
|  | 342500 | 3470000 | 0.098703 | 0.089836 | 8000 | 151500 | 0.052805 | 0.050157 |
|  | 1215000 | 6140000 | 0.197883 | 0.165194 | 284500 | 2160000 | 0.131713 | 0.116384 |
|  | 705000 | 4590000 | 0.153595 | 0.133144 | 51500 | 650000 | 0.079231 | 0.073414 |
|  | 765000 | 5020000 | 0.15239 | 0.132239 | 258000 | 1660000 | 0.155422 | 0.134515 |
|  | 30250 | 341000 | 0.08871 | 0.081481 | 105500 | 464000 | 0.227371 | 0.18525 |
|  | 152750 | 940000 | 0.1625 | 0.139785 | 308000 | 1160000 | 0.265517 | 0.209809 |
|  | 95500 | 1110000 | 0.086036 | 0.07922 | 3500 | 29500 | 0.118644 | 0.106061 |
|  | 615000 | 1580000 | 0.389241 | 0.280182 | 10000 | 188000 | 0.053191 | 0.050505 |
|  | 200000 | 890000 | 0.224719 | 0.183486 | 13500 | 161000 | 0.083851 | 0.077364 |
|  | 575000 | 3100000 | 0.185484 | 0.156463 | 680000 | 4120000 | 0.165049 | 0.141667 |
|  | 750000 | 5000000 | 0.15 | 0.130435 | 36900 | 165000 | 0.223636 | 0.182764 |
|  | 500000 | 2050000 | 0.243902 | 0.196078 | 1000 | 23000 | 0.043478 | 0.041667 |
|  | 141250 | 504000 | 0.280258 | 0.218907 | 6400 | 37750 | 0.169536 | 0.14496 |
|  | 435000 | 1650000 | 0.263636 | 0.208633 | 100 | 1100 | 0.090909 | 0.083333 |
|  | 265500 | 1250000 | 0.2124 | 0.17519 |  |  |  |  |
|  | 162500 | 1320000 | 0.123106 | 0.109612 |  |  |  |  |
|  | 920000 | 3400000 | 0.270588 | 0.212963 |  |  |  |  |
|  | 1595000 | 9030000 | 0.176633 | 0.150118 |  |  |  |  |
|  | 116000 | 394000 | 0.294416 | 0.227451 |  |  |  |  |
|  | 620000 | 3440000 | 0.180233 | 0.152709 |  |  |  |  |
|  | 1090000 | 5160000 | 0.21124 | 0.1744 |  |  |  |  |

| A909 |  |  | A909 | A909 |  |  |
| --- | --- | --- | --- | --- | --- | --- |
| ttv+sotv |  |  | sfl | sfl |  |  |
| body cfu/group of 10 flies | head/body | head/whole fly | head cfu/group of 10 flies | body cfu/group of 10 flies | head/body | head/whole fly |
| 3760000 | 0.11383 | 0.102197 | 91500 | 990000 | 0.092424 | 0.084605 |
| 260000 | 0.070385 | 0.065756 | 2700 | 35000 | 0.077143 | 0.071618 |
| 7000 | 0.214286 | 0.176471 | 121000 | 746500 | 0.16209 | 0.139481 |
| 13000 | 0.038462 | 0.037037 | 13925 | 194500 | 0.071594 | 0.066811 |
| 2030000 | 0.134729 | 0.118732 | 3850 | 53000 | 0.072642 | 0.067722 |
| 2750000 | 0.088 | 0.080882 | 0 | 35000 | 0 | 0 |
| 2770000 | 0.17148 | 0.146379 | 365000 | 2860000 | 0.127622 | 0.113178 |
| 1750000 | 0.014857 | 0.01464 | 201250 | 2100000 | 0.095833 | 0.087452 |
| 1840000 | 0.009103 | 0.009021 | 123500 | 1480000 | 0.083446 | 0.077019 |
| 2760000 | 0.127174 | 0.112825 | 770000 | 4300000 | 0.17907 | 0.151874 |
| 2640000 | 0.284091 | 0.221239 |  |  |  |  |
| 16150 | 0.111455 | 0.100279 |  |  |  |  |
|  |  |  |  |  |  |  |
