# Supplementary material for: Glycosaminoglycan Binding Facilitates Entry of a Bacterial Pathogen into Central Nervous Systems
Source: PLoS Pathog. 2011 Jun 23;7(6):e1002082. doi: 10.1371/journal.ppat.1002082 (PMC3121876; doi:10.1371/journal.ppat.1002082)
Supplement: Text S1 — Supplemental methods describing the measurement of heparan sulfate in primary mouse lung microvascular endothelial cells. (DOC) [file ppat.1002082.s007.doc]

**Supplemental Methods**

**Measurement of heparan sulfate in primary mouse lung microvascular endothelial cells.**

Cells were labeled for 24 h with 100 µCi/ml Na^35^SO_4_ in F12 nutrient mixture. Radiolabeled GAG chains were solubilized with 0.1 M NaOH for 30 min, an aliquot was removed for protein determination using the BCA protein assay (Pierce). The remaining material was adjusted to pH 7 with 10 M acetic acid and digested with a protease solution containing 1 mg/ml Pronase (Boehringer Mannheim).  After overnight incubation, the reaction mixture was diluted 5-fold with water to reduce the salt concentration to 0.1 M. The solution was applied to 0.2 ml column of DEAE-Sephacel prepared in a disposable polypropylene column. The column was washed with 20 mM sodium acetate buffer (pH 6.0) containing 0.25 M NaCl. Bound GAGs were eluted with 1 M NaCl in 20 mM sodium acetate (pH 6.0) then dialyzed into 50 mM of Ammonium Biocarbonate. The material was dried and dissolved with 200 µl of H_2_O. Chondroitin sulfate was removed by treating a sample overnight at 37 °C with 20 milliunits of chondroitinase ABC. The digested material was then put back onto a DEAE-Sephacel column and washed and eluted as described above. The [^35^S] counts in the eluent were used as a measurement of heparan sulfate in the sample.
